# Supplementary material for: Characterization and Classification of Spatial White Matter Tract Alteration Patterns in Glioma Patients Using Magnetic Resonance Tractography: A Systematic Review and Meta-Analysis
Source: Cancers (Basel). 2023 Jul 15;15(14):3631. doi: 10.3390/cancers15143631 (PMC10377290; doi:10.3390/cancers15143631)
Supplement: Supplementary file 1 [file cancers-15-03631-s001.zip › Supplementary Material S1 - Search strategies.pdf]

PubMed
(glioma [mesh] OR gliom* [tiab] OR astrocytom* [tiab] OR oligodendrogliom* [tiab] OR glioblastom* [tiab] OR GBM [tiab])

AND (diffusion magnetic resonance imaging [mesh] OR diffusion magnetic resonance imaging [tiab] OR diffusion tensor* [tiab] OR DTI [tiab] OR DWI [tiab] OR diffusion-weighted imaging [tiab] OR diffusion MRI [tiab] OR diffusion weighted MRI [tiab] OR diffusion weighted magnetic resonance [tiab] OR diffusion weighted nuclear magnetic resonance[tiab] OR tractogra* [tiab])

AND ((white matter [mesh] OR white matter [tiab] OR fiber* [tiab] OR fibre* [tiab] OR tract* [tiab] OR pathway* [tiab] OR subcortical [tiab]) AND (displac* [tiab] OR integrit* [tiab] OR alterat* [tiab] OR disrupt* [tiab] OR infiltrat* [tiab] OR destruct* [tiab] OR pattern* [tiab] OR change*[tiab] OR deviat*[tiab] OR abnormal*[tiab] OR differen*[tiab] OR discrepanc*[tiab] OR deviat*[tiab] OR devianc*[tiab] OR irregular*[tiab]))

Embase
(glioma/de OR astrocytoma/de OR oligodendroglioma/de OR glioblastoma/de OR gliom*:ti,ab,kw OR astrocytom*:ti,ab,kw OR oligodendrogliom*:ti,ab,kw OR glioblastom*:ti,ab,kw OR GBM:ti,ab,kw)

AND ('diffusion weighted imaging'/de OR 'diffusion magnetic resonance imaging':ti,ab,kw OR 'diffusion tensor*':ti,ab,kw OR DTI:ti,ab,kw OR DWI:ti,ab,kw OR 'diffusion weighted imaging':ti,ab,kw OR 'diffusion MRI':ti,ab,kw OR 'diffusion weighted MRI':ti,ab,kw OR 'diffusion weighted magnetic resonance':ti,ab,kw OR 'diffusion weighted nuclear magnetic resonance':ti,ab,kw OR tractogra*:ti,ab,kw)

AND (('white matter'/de OR 'white matter':ti,ab,kw OR fiber*:ti,ab,kw OR fibre*:ti,ab,kw OR tract*:ti,ab,kw OR pathway*:ti,ab,kw OR subcortical:ti,ab,kw) AND (displac*:ti,ab,kw OR integrit*:ti,ab,kw OR alterat*:ti,ab,kw OR disrupt*:ti,ab,kw OR infiltrat*:ti,ab,kw OR destruct*:ti,ab,kw OR pattern*:ti,ab,kw OR change*:ti,ab,kw OR deviat*:ti,ab,kw OR abnormal*:ti,ab,kw OR differen*:ti,ab,kw OR discrepanc*:ti,ab,kw OR deviat*:ti,ab,kw OR devianc*:ti,ab,kw OR irregular*:ti,ab,kw)) NOT 'conference abstract'/it AND [embase]/lim NOT ([embase]/lim AND [medline]/lim)

Cochrane Library

ID	Search
#1	MeSH descriptor: [Glioma] explode all trees
#2	(gliom* OR astrocytom* OR oligodendrogliom* OR glioblastom* OR GBM):ti OR (gliom* OR astrocytom* OR oligodendrogliom* OR glioblastom* OR GBM):ab (Word variations have been searched)
#3	#1 OR #2
#4	MeSH descriptor: [Diffusion Magnetic Resonance Imaging] explode all trees
#5	(diffusion magnetic resonance imaging OR diffusion tensor* OR DTI OR DWI OR diffusion weighted imaging OR diffusion MRI OR diffusion weighted MRI OR diffusion weighted magnetic resonance OR diffusion weighted nuclear magnetic resonance OR tractogra*):ti
#6	(diffusion magnetic resonance imaging OR diffusion tensor* OR DTI OR DWI OR diffusion weighted imaging OR diffusion MRI OR diffusion weighted MRI OR diffusion weighted magnetic resonance OR diffusion weighted nuclear magnetic resonance OR tractogra*):ab
#7	#4 OR #5 OR #6
#8	MeSH descriptor: [White Matter] explode all trees
#9	(white matter OR fiber* OR fibre* OR tract* OR pathway* OR subcortical):ti
#10	(white matter OR fiber* OR fibre* OR tract* OR pathway* OR subcortical):ab
#11	#8 OR #9 OR #10
#12	(displac* OR integrit* OR alterat* OR disrupt* OR infiltrat* OR destruct* OR pattern* OR change* OR deviat* OR abnormal* OR differen* OR discrepanc* OR deviat* OR devianc* OR irregular*):ti
#13	(displac* OR integrit* OR alterat* OR disrupt* OR infiltrat* OR destruct* OR pattern* OR change* OR deviat* OR abnormal* OR differen* OR discrepanc* OR deviat* OR devianc* OR irregular*):ab
#14	#12 OR #13
#15	#11 AND #14
#16	#3 AND #7 AND #15

Web of Science

TS=((gliom* OR astrocytom* OR oligodendrogliom* OR glioblastom* OR GBM)

AND (“diffusion magnetic resonance imaging” OR “diffusion tensor*” OR DTI OR DWI OR “diffusion weighted imaging” OR “diffusion MRI OR diffusion weighted MRI” OR “diffusion weighted magnetic resonance” OR “diffusion weighted nuclear magnetic resonance” OR tractogra*)

AND ((“white matter” OR fiber* OR fibre* OR tract* OR pathway* OR subcortical) AND (displac* OR integrit* OR alterat* OR disrupt* OR infiltrat* OR destruct* OR pattern* OR change* OR deviat* OR abnormal* OR differen* OR discrepanc* OR deviat* OR devianc* OR irregular*)))
