# Supplementary material for: Characterization and Classification of Spatial White Matter Tract Alteration Patterns in Glioma Patients Using Magnetic Resonance Tractography: A Systematic Review and Meta-Analysis
Source: Cancers (Basel). 2023 Jul 15;15(14):3631. doi: 10.3390/cancers15143631 (PMC10377290; doi:10.3390/cancers15143631)
Supplement: Supplementary file 1 [file cancers-15-03631-s001.zip › Supplementary Table S1 - MINORS.pdf]

Supplementary Table S1. MINORS methodological quality assessment.

	Bakhshi 2021	Camins 2022	Celtikci 2018	Deilami 2015	Delgado 2016	Dubey 2018	Gao 2017	Shalan 2021	Yu 2005	Zhang 2016	Zhukov 2016
**A clearly stated aim**	2	2	2	2	2	2	2	2	2	2	2
**Inclusion of consecutive patients**	2	2	2	*	2	0	0	0	2	0	0
**Prospective collection of data**	2	1	1	2	2	2	2	2	2	2	1
**Endpoints appropriate to the aim of the study**	2	2	2	2	2	2	2	2	2	2	2
**Unbiased assessment of the study endpoint**	2	0	2	0	0	0	0	0	0	0	0
**Follow-up period appropriate to the aim of the study**	2	2	2	2	2	2	2	2	2	2	2
**Loss to follow up less than 5%**	0	0	1	*	1	0	1	0	1	0	0
**Prospective calculation of the study size**	0	0	0	*	0	0	0	0	0	0	0
Total:	*12*	*9*	*12*	*8*	*11*	*8*	*9*	*8*	*11*	*8*	*7*
Additional criteria in the case of comparative study											
**An adequate control group**									2	2	
**Contemporary groups**									2	2	
**Baseline equivalence of groups**									2	2	
**Adequate statistical analysis**									1	1	
Total:									*18*	*15*	

Note: Scores meaning 0 (not reported), 1 (reported but inadequate) or 2 (reported and adequate).

* Case series.
